# Challenges and Opportunities in Accessing Surgery for Glioblastoma in Low–Middle Income Countries: A Narrative Review

**DOI:** 10.3390/cancers16162870

**Published:** 2024-08-17

**Authors:** Paolo Tini, Giovanni Rubino, Pierpaolo Pastina, Salvatore Chibbaro, Alfonso Cerase, Francesco Marampon, Sergio Paolini, Vincenzo Esposito, Giuseppe Minniti

**Affiliations:** 1 Unit of Radiation Oncology, Department of Medicine, Surgery and Neurosciences, University of Siena, 53100 Siena, Italy; g.rubino@ao-siena.toscana.it (G.R.);; 2 Unit of Neurosurgery, Department of Medicine, Surgery and Neurosciences, University of Siena, 53100 Siena, Italy; 3 Neurosurgery Department, University of Strasbourg, 67000 Strasbourg, France; 4 Unit of Neuroradiology, Azienda Ospedaliera Universitario Senese, 53100 Siena, Italy; alfonso.cerase@ao-siena.toscana.it; 5 Radiation Oncology, Policlinico Umberto I, Department of Radiological, Oncological and Pathological Sciences, “Sapienza” University of Rome, 00185 Rome, Italy; 6 Department of Neuroscience, “Sapienza” University of Rome, 00185 Rome, Italy; 7 IRCSS Neuromed, 86077 Pozzilli, Italy

**Keywords:** global disparities, access to surgery, brain tumor surgery, glioblastoma clinical impact, healthcare inequities

## Abstract

**Simple Summary:**

Glioblastoma is a highly aggressive type of brain tumor that is very difficult to treat, and surgery is crucial for improving patient survival. However, there are significant differences in access to brain tumor surgery based on factors like income, location, and available healthcare resources. People in low- and middle-income countries often struggle to receive the surgery they need due to a lack of specialized doctors, inadequate healthcare facilities, and financial challenges. As a result, patients in these regions are often diagnosed later, receive less effective treatment, and have lower survival rates compared to those in wealthier countries. This not only affects the patients but also adds economic and social burdens to their communities. The study calls for urgent actions to address these inequalities through international cooperation, better healthcare policies, and fair distribution of resources, with the goal of improving access to brain tumor surgeries for everyone, no matter where they live.

**Abstract:**

Glioblastoma: a highly aggressive brain tumor, presents substantial challenges in treatment and management, with surgical intervention playing a pivotal role in improving patient outcomes. Disparities in access to brain tumor surgery arise from a multitude of factors, including socioeconomic status, geographical location, and healthcare resource allocation. Low- and middle-income countries (LMICs) often face significant barriers to accessing surgical services, such as shortages of specialized neurosurgical expertise, limited healthcare infrastructure, and financial constraints. Consequently, glioblastoma patients in LMICs experience delays in diagnosis, suboptimal treatment, and poorer clinical outcomes compared to patients in high-income countries (HICs). The clinical impact of these disparities is profound. Patients in LMICs are more likely to be diagnosed at advanced disease stages, receive less effective treatment, and have lower survival rates than their counterparts in HICs. Additionally, disparities in access to surgical care exacerbate economic and societal burdens, emphasizing the urgent need for targeted interventions and health policy reforms to address healthcare inequities. This review highlights the importance of addressing global disparities in access to brain tumor surgery for glioblastoma through collaborative efforts, policy advocacy, and resource allocation, aiming to improve outcomes and promote equity in surgical care delivery for all glioblastoma patients worldwide.

## 1. Introduction

Glioblastoma represents one of the most challenging frontiers in oncology, marked by its high-grade malignancy and resistance to conventional treatments [1]. With an incidence rate that transcends geographical boundaries, glioblastoma imposes a substantial burden on affected individuals, families, and healthcare systems globally [2]. Among the array of therapeutic modalities available for glioblastoma, surgical intervention stands as a cornerstone in its management [3]. The ability to safely resect as much tumor tissue as possible not only alleviates symptoms but also plays a pivotal role in prolonging survival [4] and enhancing patients’ quality of life [5]. Moreover, surgery facilitates the acquisition of tissue for histopathological analysis, guiding subsequent treatment decisions and contributing to personalized therapeutic strategies [6]. Despite the critical role of surgery in glioblastoma management, access to timely and quality surgical care remains a significant challenge worldwide [7]. Disparities in healthcare infrastructure, socioeconomic status, geographical location, and cultural factors contribute to unequal distribution of surgical services, exacerbating disparities in patient outcomes. The primary objective of this review is to comprehensively examine the global disparities in access to brain tumor surgery, with a specific focus on glioblastoma and aims to identify key challenges impeding access to glioblastoma surgery and suggest opportunities for improvement in low–middle income countries (LMICs). Our review tries to fill a critical gap in the literature by providing a focused analysis of the unique barriers faced by LMICs and offering practical, context-specific recommendations for improving surgical access. This review paper is a narrative review. We chose a narrative review to provide a comprehensive and flexible synthesis of diverse studies, allowing us to capture the complexity of challenges and opportunities in LMICs with the aim of offering a cohesive understanding and actionable insights tailored to resource-limited settings and integrating how solutions in high-income countries can be adapted to LMICs.

## 2. Methodology

We used a comprehensive methodology to explore global disparities in access to brain tumor surgery for glioblastoma in LMICs. The methodology encompasses an extensive literature search, inclusion and exclusion criteria, data extraction, and thematic analysis to synthesize finding and identify key challenges and opportunities.

### 2.1. Literature Search

- An extensive search of electronic databases (PubMed, MEDLINE, Embase, Scopus, Web of Science, and Google Scholar) was conducted.
- Keywords and medical subject headings (MeSH) terms used for the search included variations of “glioblastoma”, “brain tumor surgery”, “access”, “disparities”, and “global”.
- The search strategy was tailored to identify peer-reviewed articles and reviews published in English from inception to the present date.

### 2.2. Inclusion and Exclusion Criteria

- Articles were included if they addressed disparities in access to glioblastoma surgery on a global scale, focusing on LMICs, examined factors contributing to these disparities, and discussed potential strategies for improving access in LMICs.
- Exclusion criteria encompassed studies focusing on non-glioblastoma brain tumors or non-surgical interventions, articles not available in full text or the English language, and articles in papers which did not adopt a peer-review process to publish.

### 2.3. Data Extraction

- Relevant data, including study objectives, methodologies, key findings, and recommendations, were extracted from selected articles.
- Emphasis was placed on identifying patterns, trends, and variations in access to glioblastoma surgery across different regions, healthcare systems, and socio-economic contexts, with a focus on LMICs.

### 2.4. Thematic Analysis

- Extracted data were subjected to thematic analysis [8] to identify recurring themes, common barriers, and facilitators influencing access to glioblastoma surgery globally, and in LMICs.
- Themes were iteratively refined through an inductive process, allowing for the identification of nuanced perspectives and emergent issues.

### 2.5. Synthesis and Interpretation

- The synthesis of findings involved organizing extracted data into coherent narratives, elucidating the complex interplay of factors shaping access to glioblastoma surgery.
- The interpretation of findings was guided by emerged common themes related to healthcare disparities, equity, and access to surgical care.

## 3. Epidemiology and Burden of Glioblastoma

Glioblastoma represents the most common and aggressive primary malignant brain tumor in adults [2]. The incidence of glioblastoma varies globally, with rates typically ranging from two to three cases per 100,000 individuals per year [9]. However, incidence rates may vary by geographical region, age, and other demographic factors [10]. Despite advances in diagnostic imaging and treatment modalities, the incidence of glioblastoma appears to be increasing in some regions [11], underscoring the need for continued surveillance and research efforts to elucidate contributing factors. Glioblastoma is characterized by its infiltrative growth pattern, high proliferative rate, and resistance to treatment [12]. Histologically, glioblastomas are classified as Grade IV tumors according to the World Health Organization (WHO) classification system [13], exhibiting cellular and molecular heterogeneity that complicates treatment approaches. Despite multimodal therapies incorporating surgery, radiation, and chemotherapy, the prognosis for glioblastoma remains poor, with a median survival of approximately 12 to 16 months from the time of diagnosis; disease recurrence is almost inevitable and long-term survival rates remain dismally low [14,15]. The burden of glioblastoma extends beyond the clinical realm, exerting profound physical, emotional, and socioeconomic impacts on patients, their families, and healthcare systems [16,17]. Moreover, the financial costs of glioblastoma treatment, including medical expenses, lost productivity, and supportive care services, can impose substantial economic burdens on patients and their families [18]. At the healthcare system level, glioblastoma presents significant challenges in resource allocation, delivery, and quality of care. Its complex treatment requires multidisciplinary teams, including neurosurgeons, neuro-oncologists, radiation oncologists, and supportive care specialists. However, disparities in access to specialized care, diagnostic resources, and treatment facilities may compromise the delivery of optimal care to glioblastoma patients, exacerbating health inequities and disparities in outcomes [19], which appears to be the case in LMICs.

## 4. Current Status of Access to Glioblastoma Tumor Surgery Worldwide

**Figure 1:**
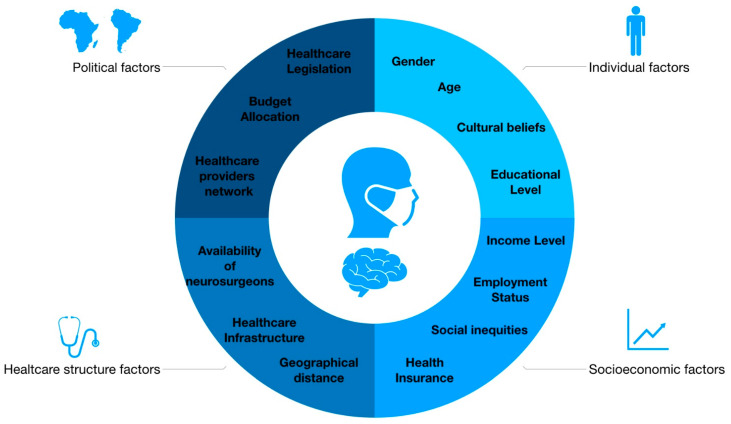
Intertwined factors determining access to glioblastoma surgery.

Access to healthcare is a fundamental human right and a key determinant of health outcomes. The concept of access encompasses multiple dimensions, including geographical, financial, cultural, and structural factors, all of which influence an individual’s ability to obtain timely and appropriate medical care [20]. In the context of brain tumor surgery, access plays a pivotal role in determining patient outcomes and quality of life [21,22,23,24,25,26,27]. Despite advancements in neurosurgical techniques and the proliferation of specialized centers of excellence, access to brain tumor surgery remains unevenly distributed globally [28]. Disparities in access to surgical care for brain tumors are evident across different regions, income levels, and healthcare systems, potentially contributing to variations in patient outcomes and survival rates [29]. Geographical factors, such as the availability of healthcare facilities, transportation infrastructure, and geographic remoteness [30]. Financial barriers such as out-of-pocket expenses, insurance coverage, and care affordability significantly impact access to brain tumor surgery [31,32]. Cultural beliefs, language barriers, and healthcare-seeking behaviors shape individuals’ willingness and ability to access brain tumor surgery [33,34]. Structural factors within healthcare systems, including the availability of healthcare providers, referral pathways, waiting times, and healthcare infrastructure, impact access to brain tumor surgery [35,36,37]. All these factors overlap and intertwine, collectively shaping the varying levels of access to brain tumor surgery (Figure 1).

In particular, access to glioblastoma surgery is influenced by a multitude of factors that intersect at individual, social, and systemic levels [38]. Understanding these multifactorial determinants is critical for addressing access disparities and improving outcomes for patients with glioblastoma. Individual factors such as age, gender, education and awareness about glioblastoma, its symptoms, and available treatment options, as well as cultural beliefs, values, and attitudes towards the disease, can significantly influence decisions regarding treatment modalities, acceptance of surgical interventions, and adherence to postoperative care regimens [39,40]. Patients with higher levels of literacy and health awareness are more likely to access appropriate surgical therapeutic interventions [39]. Socioeconomic factors, including income level, employment status, access to health insurance, social and community networks, and experiences of discrimination and social inequities, play a crucial role in determining access to glioblastoma surgery [41,42,43,44]. These elements collectively impact the ability of patients to seek and receive necessary care.

Systemic factors, particularly healthcare infrastructure and geographical distances, significantly affect access to glioblastoma surgery [44,45]. Regions equipped with advanced neurosurgical centers, state-of-the-art imaging facilities, and multidisciplinary care teams are better positioned to provide timely surgical interventions. Additionally, health policy and organizational factors such as healthcare legislation, government funding and budget allocation, health insurance regulations, and the structure of healthcare provider networks also play a pivotal role [46,47,48]. In summary, access to glioblastoma surgery is influenced by a complex interaction of individual, social, and systemic factors. Identifying and addressing barriers at these levels is essential for ensuring equitable access to surgical care and improving outcomes for patients with glioblastoma

## 5. Disparities and Access to Glioblastoma Surgery and Their Impact on Survival Patients

This section synthesizes findings from recent studies to elucidate the impact of individual, socioeconomic, structural, and systemic factors on access to glioblastoma surgery and its consequent effects on survival rates (Table 1).

### 5.1. Socioeconomic Disparities

Socioeconomic status (SES) significantly influences access to glioblastoma surgery and subsequent treatments. Rivera Perla et al. [42] utilized the area deprivation index (ADI) to study patients with glioblastoma, revealing that those in more disadvantaged areas (ADI 34–100%) had decreased odds of receiving gross total resection (aOR 0.43) and were less likely to undergo chemotherapy, radiation, or participate in clinical trials compared to their less disadvantaged counterparts (ADI 0–33%). Similarly, a comprehensive review by Gorenflo et al. [43] highlighted that higher area-level SES is positively correlated with both glioblastoma incidence and prognosis in the United States, underscoring the necessity of large study populations to assess SES and glioblastoma prognosis effectively. In Italy, a study by Tosoni et al. [49] confirmed that SES impacts clinical outcomes even within a National Health Service providing universal healthcare. Higher-income patients had significantly better overall survival (HR = 0.641) after adjusting for various factors, including age and surgical extent. These findings collectively indicate that socioeconomic disparities remain a critical determinant of glioblastoma prognosis, despite efforts to provide equitable healthcare.

### 5.2. Racial and Ethnic Disparities

Racial and ethnic disparities further compound the inequities in glioblastoma treatment. Ostrom et al. [50] analyzed data from the National Cancer Database, finding that Black non-Hispanics and Hispanics were less likely to receive radiation and chemotherapy compared to White non-Hispanics. The study also found delays in the initiation of these treatments for minority groups, significantly affecting survival outcomes. This evidence underscores the urgent need for targeted interventions to address racial and ethnic disparities in glioblastoma care.

### 5.3. Impact of Insurance Status

Insurance status is another pivotal factor influencing access to glioblastoma surgery and survival. Chandra et al. [46] reported that Medicaid patients incurred 30% higher overall hospital costs for surgery and had significantly longer hospital stays compared to non-Medicaid patients. These patients also presented with larger tumors and had poorer preoperative and postoperative functional scores, resulting in shorter median overall survival (10.7 months for Medicaid vs. 15.8 months for privately insured). Similarly, Brown et al. [47] found that Medicaid and uninsured patients were less likely to receive surgery, radiation, and chemotherapy, which independently impacted survival rates. Ensuring adequate access to care for all patients, regardless of insurance status, is critical for optimizing survival outcomes, especially as therapies continue to advance.

### 5.4. Healthcare Infrastructure and Hospital Type

The type of healthcare facility also plays a significant role in the management and outcomes of glioblastoma patients. Brandel et al. [48] investigated the treatment of glioblastoma at safety-net hospitals, which cater to a disproportionate number of underserved patients. Patients at high-burden hospitals were less likely to receive standard-of-care therapies and exhibited higher short- and long-term mortality rates compared to those treated at low-burden hospitals.

The studies summarized herein reveal a complex interplay of socioeconomic, racial, insurance, and infrastructural factors that contribute to global disparities in access to glioblastoma surgery and survival outcomes.

## 6. Low- and Middle-Income Countries: Situations

Low- and middle-income countries (LMICs) face significant challenges in providing equitable access to glioblastoma surgery, resulting in stark disparities compared to high-income countries (HICs) [51,52,53,54,55]. The management of glioblastoma is heavily dependent on timely surgical intervention followed by adjuvant therapies. However, resource constraints, inadequate healthcare infrastructure, and systemic barriers in LMICs contribute to delayed diagnoses, suboptimal treatments, and poorer patient outcomes, as follows:

*Resource constraints:* LMICs often suffer from limited healthcare budgets that prioritize primary care over specialized treatments. This leads to underinvestment in neurosurgical facilities, essential surgical instruments, neuroimaging technologies, and perioperative medications. Such resource limitations severely hinder the ability to deliver safe and effective surgical interventions for glioblastoma patients [51,55]. The high costs associated with glioblastoma treatment, including unregulated expenses for radiation and chemotherapy, often impose severe financial burdens on patients. This financial barrier is compounded by the lack of comprehensive health insurance, frequently resulting in treatment discontinuation

*Lack of expertise:* There is a critical shortage of neurosurgeons and specialized healthcare professionals in LMICs [56,57,58]. The uneven distribution of trained professionals, coupled with limited training opportunities, exacerbates these challenges. In many rural and underserved areas, the absence of qualified neurosurgeons delays diagnosis and treatment, contributing to advanced disease stages at presentation

*Infrastructure deficiencies:* Inadequate neurosurgical healthcare infrastructure is a significant barrier in LMICs [57,58]. Many hospitals lack dedicated neurosurgical units and intensive care facilities necessary for managing postoperative complications. Issues such as unreliable electricity, lack of sterilization equipment, and insufficient infection control measures further compromise patient safety and surgical outcomes.

*Systemic barriers:* Bureaucratic inefficiencies, fragmented healthcare systems, and regulatory hurdles impede timely access to glioblastoma surgery. Complex referral processes and long waiting times delay treatment initiation, worsening patient prognosis [57]. Limited coordination between primary care providers and specialized centers results in suboptimal patient management.

## 7. Opportunities for Improvement

**Figure 2:**
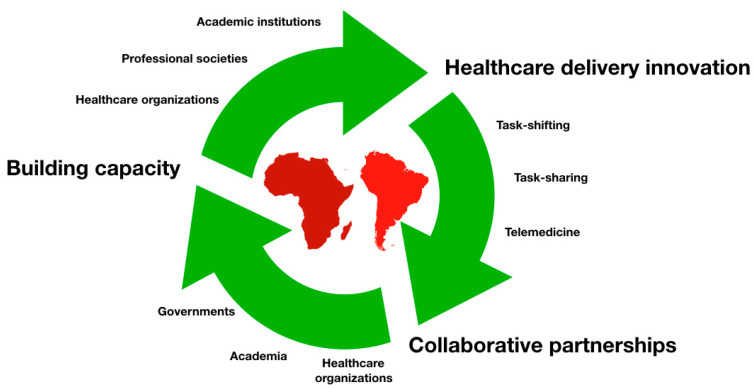
Opportunities to improve access to glioblastoma surgery in low–middle income countries.

By analyzing various authors’ suggestions for possible improvements in low-income areas, we found the following (Figure 2):

Healthcare delivery innovation: Innovative healthcare delivery models, such as task-shifting and task-sharing, can enhance surgical capacity in resource-constrained settings [59]. Training non-physician healthcare providers in basic neurosurgical skills and integrating neurosurgical services into primary care settings can facilitate the early detection, referral, and management of glioblastoma cases. Telemedicine and digital health technologies offer significant potential to improve access to surgery in remote areas [60]. Teleconsultation platforms enable remote diagnosis, treatment planning, and follow-up care, reducing the need for extensive travel. Telementoring programs promote knowledge exchange between neurosurgeons in high-resource and low-resource settings, enhancing local surgical capacity and outcomes

Capacity-building initiatives: Investing in healthcare workforce training and professional development is crucial for building neurosurgical capacity in LMICs [61,62,63]. Collaborative initiatives involving academic institutions, professional societies, and healthcare organizations can facilitate skills transfer and mentorship for neurosurgeons and allied health professionals. Fellowship programs, short-term training courses, and surgical missions led by international experts provide valuable hands-on training opportunities.

Collaborative partnerships: Collaborative partnerships between governments, healthcare organizations, academia, and non-governmental organizations (NGOs) are essential to improving access to glioblastoma surgery globally [64,65,66]. Multilateral collaborations, such as the World Health Organization’s Global Neurosurgery Initiative, facilitate knowledge sharing, advocacy, and policy dialog. Public–private partnerships and philanthropic initiatives can mobilize resources, expertise, and technology to support capacity-building efforts in underserved regions.

## 8. Discussion

Addressing the challenges of accessing glioblastoma surgery in low-resource settings demands a comprehensive approach that goes beyond simply increasing resources. It requires strengthening healthcare infrastructure, enhancing specialized expertise, and addressing systemic barriers to care. Investments in healthcare workforce training, infrastructure development, and broader health system improvements are crucial to narrowing the gap in surgical care outcomes between high- and low-income countries. However, this review has several limitations that must be acknowledged. The inclusion of only experimental studies published in English may have led to a selection bias, excluding relevant research from non-English sources or gray literature. Additionally, the diversity in study designs and methodologies among the included studies may affect the consistency and generalizability of the findings. The review’s focus on specific regions within low- and middle-income countries may not fully capture the diverse healthcare contexts across all such settings, and inconsistencies in reporting across studies could impact the clarity of the synthesized findings. Furthermore, the rapidly evolving healthcare policies and resources in these countries mean that the findings may not reflect the most recent developments. The review also tends to emphasize provider and systemic perspectives, with limited exploration of patient experiences, which could offer additional insights into the challenges and opportunities in accessing glioblastoma surgery.

## 9. Conclusions

To improve access to glioblastoma surgery in low-resource settings, a multi-dimensional strategy is essential. This includes addressing resource limitations, enhancing healthcare infrastructure, and developing specialized expertise, while also tackling systemic barriers to care. Collaborative efforts involving governments, international organizations, academic institutions, and non-governmental organizations are vital for overcoming the challenges faced by glioblastoma patients in these regions. Despite the review’s limitations, it underscores critical areas for improvement and highlights the importance of equitable access to surgical interventions for all individuals affected by this devastating disease. Future research should aim to address the gaps identified in this review, particularly the need for more comprehensive data on patient outcomes and the inclusion of patient perspectives, to better inform strategies and interventions that can improve surgical access and outcomes for glioblastoma patients globally.

## Figures and Tables

**Table 1:** Key factors contributing to disparities in access to glioblastoma surgery and their impact on patient survival.

Factor	Findings	Impact on Access and Survival
Socioeconomic Disparities	- Lower area deprivation index (ADI) associated with reduced odds of receiving gross total resection [40,42].	- Decreased likelihood of undergoing chemotherapy, radiation, or participating in clinical trials [40,42].
	- Higher Socioeconomic Status (SES) linked to better glioblastoma incidence and prognosis [41,43].	- Higher-income patients had significantly better overall survival (HR = 0.641) [47,49].
Racial and Ethnic Disparities	- Black non-Hispanics and Hispanics less likely to receive radiation and chemotherapy [48,50].	- Delays in treatment initiation for minority groups, negatively affecting survival outcomes [48,50].
Impact of Insurance Status	- Medicaid patients incurred 30% higher hospital costs and had longer hospital stays [44,46].	- Medicaid patients had shorter median overall survival (10.7 months vs. 15.8 months for privately insured) [44,46].
	- Medicaid and uninsured patients less likely to receive surgery, radiation, and chemotherapy [45,47].	- Reduced access to treatments independently impacted survival rates [45,47].
Healthcare Infrastructure and Hospital Type	- Patients at safety-net hospitals less likely to receive standard-of-care therapies [48].	- Higher short- and long-term mortality rates for patients at high-burden hospitals compared to low-burden hospitals [48].
